# Expression and Function of Variants of Human Catecholamine Transporters Lacking the Fifth Transmembrane Region Encoded by Exon 6

**DOI:** 10.1371/journal.pone.0011945

**Published:** 2010-08-05

**Authors:** Chiharu Sogawa, Chieko Mitsuhata, Kei Kumagai-Morioka, Norio Sogawa, Kazumi Ohyama, Katsuya Morita, Katsuyuki Kozai, Toshihiro Dohi, Shigeo Kitayama

**Affiliations:** 1 Department of Dental Pharmacology, Okayama University Graduate School of Medicine, Dentistry and Pharmaceutical Sciences, Okayama, Japan; 2 Department of Pediatric Dentistry, Hiroshima University Graduate School of Biomedical Sciences, Hiroshima, Japan; 3 Department of Dental Pharmacology, Hiroshima University Graduate School of Biomedical Sciences, Hiroshima, Japan; 4 RI Research Center, Okayama University Dental School, Okayama, Japan; University of Cambridge, United Kingdom

## Abstract

**Background:**

The transporters for dopamine (DAT) and norepinephrine (NET) are members of the Na^+^- and Cl^−^-dependent neurotransmitter transporter family SLC6. There is a line of evidence that alternative splicing results in several isoforms of neurotransmitter transporters including NET. However, its relevance to the physiology and pathology of the neurotransmitter reuptake system has not been fully elucidated.

**Methodology/Principal Findings:**

We found novel isoforms of human DAT and NET produced by alternative splicing in human blood cells (DAT) and placenta (NET), both of which lacked the region encoded by exon 6. RT-PCR analyses showed a difference in expression between the full length (FL) and truncated isoforms in the brain and peripheral tissues, suggesting tissue-specific alternative splicing. Heterologous expression of the FL but not truncated isoforms of DAT and NET in COS-7 cells revealed transport activity. However, immunocytochemistry with confocal microscopy and a cell surface biotinylation assay demonstrated that the truncated as well as FL isoform was expressed at least in part in the plasma membrane at the cell surface, although the truncated DAT was distributed to the cell surface slower than FL DAT. A specific antibody to the C-terminus of DAT labeled the variant but not FL DAT, when cells were not treated with Triton for permeabilization, suggesting the C-terminus of the variant to be located extracellulary. Co-expression of the FL isoform with the truncated isoform in COS-7 cells resulted in a reduced uptake of substrates, indicating a dominant negative effect of the variant. Furthermore, an immunoprecipitation assay revealed physical interaction between the FL and truncated isoforms.

**Conclusions/Significance:**

The unique expression and function and the proposed membrane topology of the variants suggest the importance of isoforms of catecholamine transporters in monoaminergic signaling in the brain and peripheral tissues.

## Introduction

Neurotransmitter transporters accumulate extracellular neurotransmitters released from nerve terminals to maintain synaptic clearance, thereby controlling the fine-tuning of neurotransmission [1]. Psychostimulants including cocaine and amphetamines exert their pharmacological effects by acting on monoamine neurotransmitter transporters for dopamine (DAT), norepinephrine (NET) and serotonin (SERT) [2], [3]. DAT, NET, and SERT, along with transporters for GABA and glycine, are Na^+^- and Cl^−^-dependent neurotransmitter transporters, having 12 hydrophobic transmembrane domains (TMDs) and intracellular N- and C-termini [4], [5].

There is increasing evidence that neurotransmitter transporters are not constitutively expressed at nerve endings, but rather, dynamically regulated by various cellular mechanisms. One such mechanism could be alternative splicing. We and others have reported various NET splice variants in different species including rats, cows and humans [see ref. 6 for review]. However, there is no evidence for the occurrence of DAT isoforms produced by alternative splicing.

Recently, Miller et al. [7] reported a variant of monkey NET, generated by alternative splicing in the region encoded by exon 6. They reported that when expressed in COS-7 cells, the variant failed to reveal any transport activity. However, few details were given about the structure, function and expression of this mutant. Skipping exon 6 causes the nucleotide sequence to shift in frame, resulting in evasion of nonsense mediated decay (NMD) operating to eliminate aberrant RNA [8]. This isoform of monkey NET was predicted to lack the 5th TMD, leading to a potentially unique membrane topology.

Recently, Yamashita et al. [9] elucidated the structure of a leucine transporter (LeuT), a bacterial homologue of the Na^+^- and Cl^−^-dependent neurotransmitter transporters, using X-ray crystallography. These results confirmed the previously predicted 12 TMD structure, and provided new structural details. Furthermore, they suggested the dimerization of LeuT, with the 9th and 12th TMDs interacting [9]. A similar conclusion was reached for mammalian Na^+^- and Cl^−^-dependent neurotransmitter transporters including DAT based on biochemical and molecular biological evidence [10]. We also reported that variants of rat NET produced by alternative splicing in the C-terminal region had a dominant negative effect on the functional expression of not only wild-type NET but also wild-type DAT [11]. Furthermore, Hahn et al. [12] recently demonstrated that a mutation in the human NET gene associated with orthostatic intolerance disrupted the surface expression of mutant and wild-type transporters, resulting from oligomerization as a potential mechanism of the dominant negative effect. Given these results, it is difficult to speculate on the structure of the isoform lacking the 5th TMD, and how splice variants lacking exon 6 might exhibit a dominant negative effect via interaction with wild-type DAT.

We initially identified isoforms of human NET (hNET) and DAT (hDAT) as counterparts of the monkey NET variant missing exon 6, in various tissues, since both DAT and NET are known to exist not only in the central nervous system but also in peripheral tissues, e.g. lymphocytes and gut for DAT [13], [14] and placenta for NET [15]. Thus, we further explored the mechanism of the functional expression of the novel hDAT and hNET isoforms. The current study demonstrated the unique expression and function and proposed a membrane topology of the hDAT and/or hNET variants that might contribute to our understanding of the importance of alternative splicing for monoamine neurotransmitter transporters.

## Results

### Identification and characterization of variants of human catecholamine transporters

The screening of hNET cDNAs by RT-PCR in SK-N-SH cells [16] resulted in the identification of additional hNET variants, one of which lacked the region encoded by exon 6. We designated this clone hNETΔEX6. Since a monkey NET variant missing exon 6 has been reported, we explored the possible occurrence of counterparts among hDAT and hNET.

An initial search of the EST database identified human NET but not DAT transcripts, which skipped the exon 6 region (e.g. BE260309, BE314831, etc). We could not find any candidates for variants of the DAT and NET transcripts in rat and mouse EST database. Since the organization of the hDAT and hNET genes has been well characterized [17], [18] (Fig. S1A), we compared the nucleotide sequences of the hDAT and hNET genes at exon 6 and nearby introns predicting different splicing between hDAT and hNET. A search with the web-based program ESEfinder, which predicts putative binding sites (exonic splicing enhancer elements, ESE) six to eight nucleotides long for the SR proteins SF2/ASF, SC35, SRp40, and SRp55 in any gene of interest [19], uncovered ESE motifs for all these proteins in the nucleotide sequences of hNET and hDAT cDNAs, although the hNET sequence lacked the SF2/ASF motif. These results together with a weak consensus sequence of the pyrimidine-rich intronic 5′-region at the junction in the hNET but not hDAT gene (Fig. S1B) suggested an increased occurrance of alternative splicing at exon 6 for hNET rather than hDAT.

**Figure 1:**
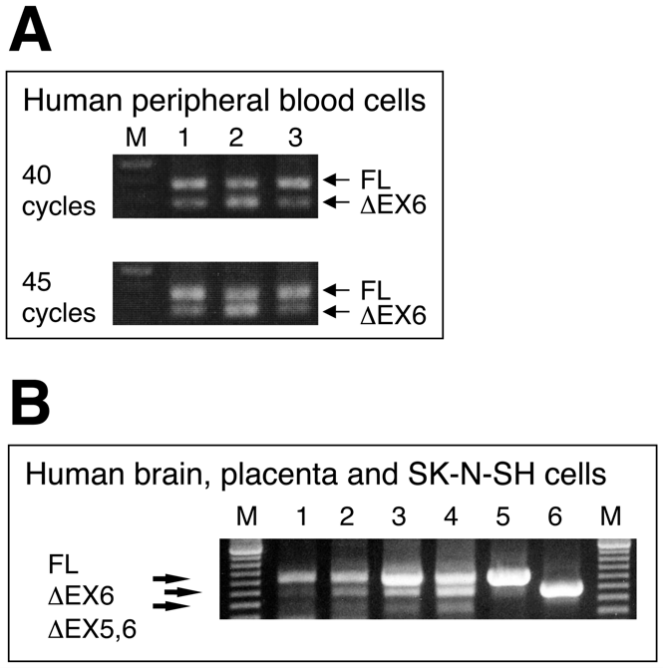
RT-PCR analysis of the human dopamine and norepinephrine transporter variants produced by alternative splicing. **A.** Total RNA from white blood cells of three healthy volunteers (lane 1–3) was used to synthesize first strand cDNA for analyzing the expression of a variant of the dopamine transporter by RT-PCR and agarose gel electrophoresis. **B.** Expression of variants of the norepinephrine transporter was examined in adult whole brain (lane 1), fetal whole brain (lane 2), placenta (lane 3) and SK-N-SH cells (lane 4) with controls (FL hNET, lane 5 and hNETΔEX6, lane 6). The data represent a typical example from one experiment followed by at least 2 additional experiments with similar results. M; DNA marker of 100 bp ladders.

For human DAT, we found a counterpart of the splice variant in peripheral white blood cells (Fig. 1A), where wild-type DAT mRNA has been found [13]. Changes in nucleotide sequence of the variant, designated hDATΔEX6, were in frame due to loss of the region encoded by exon 6. However, we could not detect the variant in total RNA from human brain tissue, or in a cDNA library from human substantia nigra, both obtained from commercial sources, under any PCR conditions tested (data not shown). Also, in preliminary experiments, attempts to amplify the splice variant from the brain tissue of patients with Parkinson's disease failed (Fig. S2). The presence of DAT mRNA and protein in the murine bowel, possibly in enteric dopaminergic neurons, has been reported [14], but we did not examine the variant there.

For human NET, variant mRNA lacking the region encoded by exon 6 was detecteded in placenta and fetal brain by RT-PCR (Fig. 1B). Changes in the nucleotide sequence of the variant, designated hNETΔEX6, were in frame due to loss of the region encoded by exon 6. In addition, we found another variant lacking the region encoded by exons 5 and 6, which causes a frame shift, resulting in a stop codon in the region encoded by exon 7. hNETΔEX6 mRNA was also detected in the adrenals and fetal brain but not in the adult brain, suggesting a tissue-specific and development-dependent splicing of hNET transcripts.

### Functional characterization of human DAT and NET variants

**Figure 2:**
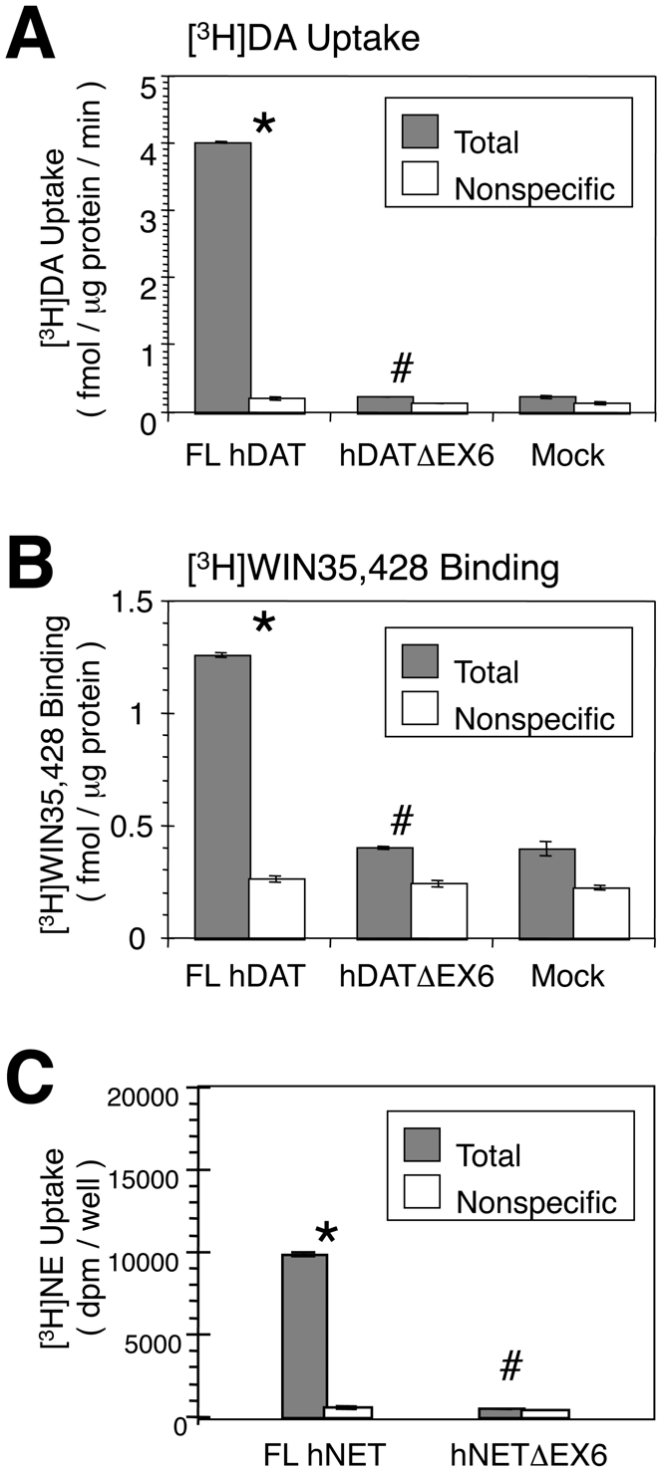
Functional characterization of the DAT and NET variants heterologously expressed in COS-7 cells. **A, B.** COS-7 cells were transfected with full length (FL) hDAT, hDATΔEX6, and empty vector pcDNA3 (Mock), and then subjected to a [^3^H]dopamine (DA) uptake assay (A) and [^3^H]WIN35,428 binding assay (B) in parallel. Two days after transfection, cells were incubated with 10 nM [^3^H]DA at 37°C for 6 min (A) or with 2 nM [^3^H]WIN35,428 at 4°C for 2 h (B) in the absence (Total) or presence (Nonspecific) of 100 µM cocaine (A) or 1 mM DA (B), respectively. **C.** COS-7 cells were transfected with FL hNET and hNETΔEX6, and then subjected to a [^3^H]NE uptake assay. Nonspecific uptake was determined in the presence of 10 µM nisoxetine. Values represent the mean ± SEM for 3 experiments each performed in triplicate. Statistical analyses were performed using an analysis of variance (ANOVA) with pair-wise comparisons by the Bonferroni method. *P<0.05 vs Nonspecific and/or Mock, #P<0.05 vs FL-DAT or FL-NET.

Figure 2A shows the uptake of [^3^H]DA in COS-7 cells transiently expressing the full-length (FL) hDAT and the splice variant, hDATΔEX6. While the cells expressing FL hDAT revealed a robust uptake of [^3^H]DA, hDATΔEX6 failed to induce any uptake of [^3^H]DA. Furthermore, no specific binding of the cocaine analogue WIN35,428 was found in COS-7 cells expressing hDATΔEX6 (Fig. 2B).

Similarly, hNETΔEX6 did not transport [^3^H]NE, when transiently expressed in COS-7 cells (Fig. 2C).

### Subcellular distribution of human DAT and NET variants

**Figure 3:**
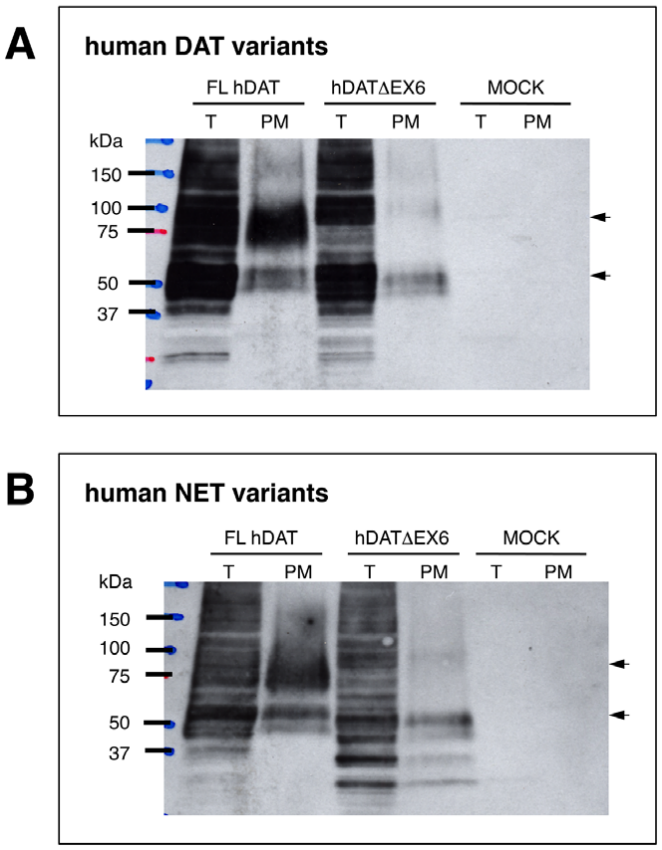
Biochemical analysis of the cell surface expression of hDAT and hNET variants in the transfected COS-7 cells. COS-7 cells transfected with FL hDAT and hDATΔEX6 (**A**), or FL hNET and hNETΔEX6 (**B**) were incubated with sulfo-NHS-SS-biotin for 60 min at 4°C, and then processed for the isolation of biotinylated DAT or NET proteins in the plasma membrane (PM) or total fraction (T). Western blotting with hDAT- or hNET-specific antibody demonstrated the cell surface expression of 80–75 kDa and 50–45 kDa hDATΔEX6 or 80 kDa and 55–50 kDa hNETΔEX6 in association with FL hDAT or FL hNET, respectively. Data represent a typical result of at least 3 repetitive experiments.

Immunoblots of COS-7 cells transiently expressing FL hDAT revealed both 80–85 kDa and 50 kDa bands in the total extract (Figure 3A). The immunoreactivity to anti-hDAT antibody of NeutrAvidin-precipitates from biotinylated extracts was increased, with the 80–85 kDa band more intense than the 50 kDa band, suggesting the cell surface expression of mature and, to a lesser extent, immature forms of FL hDAT. Loss of the region encoded by exon 6 caused a marked reduction in levels of mature protein (80–85 kDa band) with a greater increase in levels of immature protein (50 kDa band) in total fractions, in association with a striking loss of mature but not immature protein in cell surface fractions (Figure 3A). hDATΔEX6 looked slightly larger than FL hDAT in the biotinylated fraction. One explanation for this apparent discrepancy is that the conformational change due to truncation at TM5 reduces the mobility of hDATΔEX6 in the gel. Alternatively, hDATΔEX6 may be glycosylated differently due to the conformational change, resulting in a larger molecular weight. Further study is needed to clarify this issue.

Cell surface biotinylation and Western blot analyses in COS cells transfected with FL and truncated hNET cDNAs using a specific antibody to hNET revealed results similar to those observed for hDAT (Fig. 3B).

**Figure 4:**
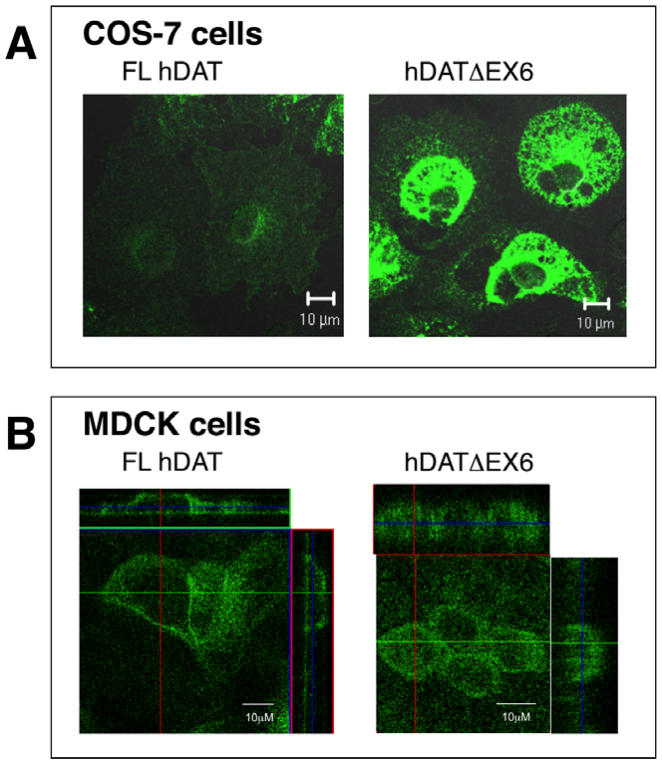
Immunofluorescence imaging of the FL hDAT and hDATΔEX6 in the transfected COS-7 and MDCK cells. **A.** COS-7 cells were transiently transfected with FL hDAT and hDATΔEX6. After 2 days, the subcellular distribution of the DAT variants was assessed by confocal microscopy with immunological detection using a human DAT-specific antibody. B. The subcellular localization of hDAT variants was examined in MDCK cells stably expressing FL hDAT and hDATΔEX6.

Immunocytochemical detection with confocal microscopy demonstrated the transient expression of FL hDAT at the surface of COS-7 cells and an intracellular distribution (Fig. 4A). In contrast, hDATΔEX6 was mainly located in the cytosolic compartment with less expression at the cell surface (Fig. 4A). We further analyzed the subcellular distribution of hDAT variants in MDCK cells stably expressing FL hDAT and hDATΔEX6. The cell surface expression of FL hDAT and to a lesser extent, hDATΔEX6, was observed (Fig. 4B). A Z-axis analysis revealed that hDAT localized apically (Fig. 4B) consistent with a previous report for DAT [20], which was in contrast to the basolatelal distribution of hNET we observed previously [21].

**Figure 5:**
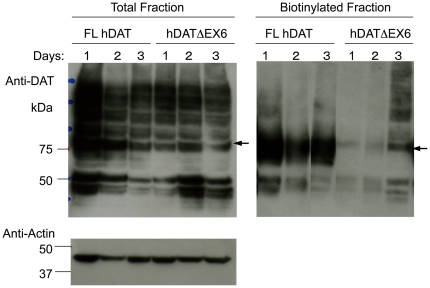
Time-course of the cell surface expression of human DAT variants in COS-7 cells. COS-7 cells were transiently transfected with FL DAT or hDATΔEX6. After 1, 2, and 3 days, cells were incubated with sulfo-NHS-SS-biotin for 120 min at 4°C, and then processed for the isolation of biotinylated hDAT in the plasma membrane. Western blotting with the hDAT-specific antibody demonstrated the cell surface expression of 80 kDa and 50 kDa hDATΔEX6 in association with FL hDAT. The data represent a typical result from one experiment followed by 2 additional experiments with similar results.

We further analyzed the time-course of the cell surface expression of hDAT variants. Fig. 5 shows immunoblots of total and biotinylated extracts from COS cells expressing FL hDAT and hDATΔEX6 at days 1, 2 and 3 after transfection. The expression of both total and biotinylated FL hDAT peaked 1 day after transfection. In contrast, the cell surface expression of hDATΔEX6 increased gradually, reaching a maximum at 3 days, while the total protein level was constant after transfection. During these periods, no transport activity was found in the cells transfected with hDATΔEX6 cDNA (data not shown).

### Membrane topology of the human DAT variants

A hydropathic analysis of hDATΔEX6 revealed 11 putative transmembrane domains (TMDs), suggesting a membrane topology different from that of FL hDAT. If TMDs are inserted behind the truncation, the C-terminus could be located extracellulary. To explore this possibility, an immunocytochemical analysis with confocal microscopy was performed using two specific antibodies, one recognizing the second extracellular region (anti-hDAT-EL2 antibody) and the other recognizing the intracellular C-terminus (anti-hDAT-Ct antibody).

**Figure 6:**
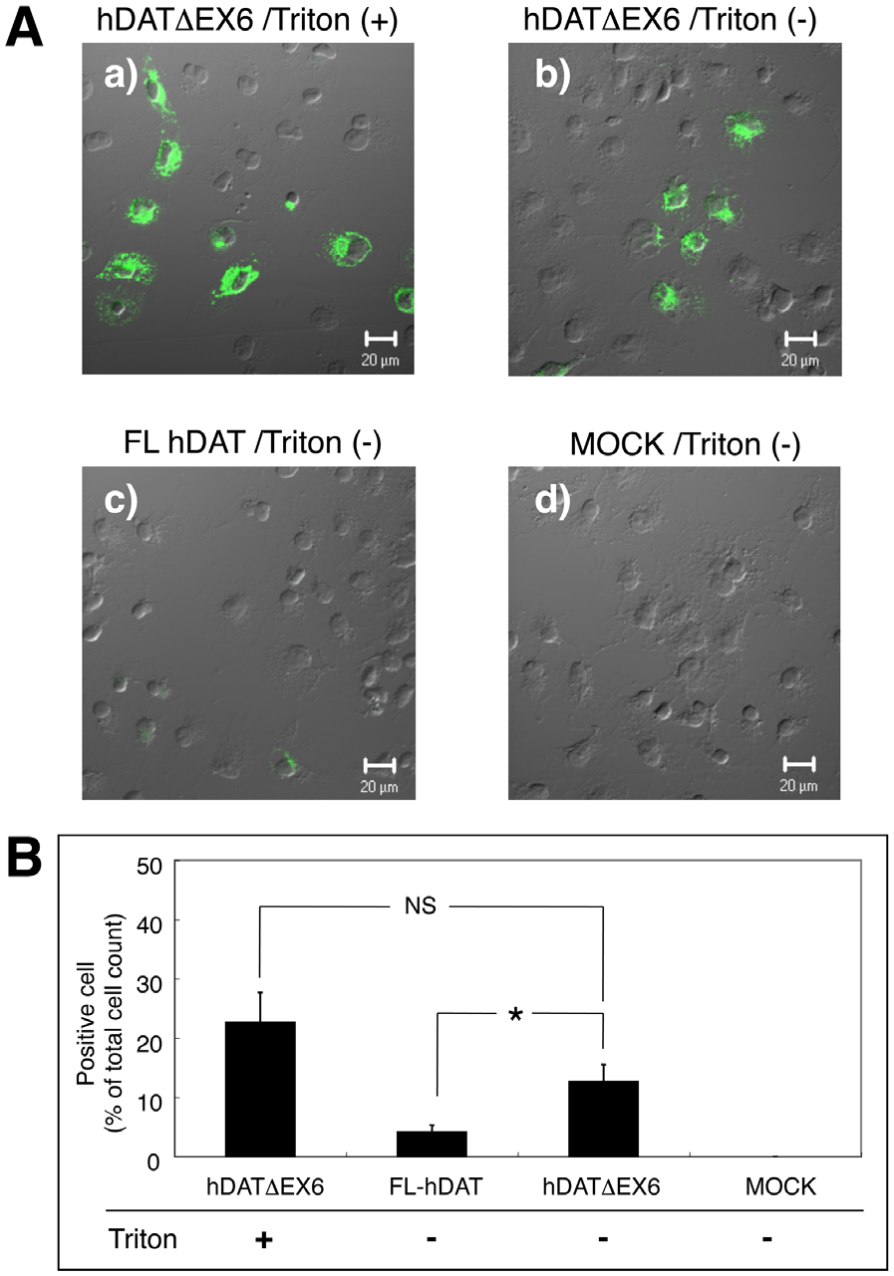
Immunofluorescence detection of the hDAT variants using anti-hDAT-EL2 antibody in COS-7 cells treated with Triton. **A.** Merged pictures of differential interference contrast and fluorescence images taken from COS-7 cells transiently transfected with hDATΔEX6 (a, b), FL hDAT (c) or empty vector pcDNA3 (Mock, d). Two days after transfection, cells were treated with (a) or without (b–d) 0.25% Triton X-100 for 5 min, and then examined for the expression of DAT variants by confocal microscopy with immunological detection using an anti-hDAT-Ct antibody. **B.** Quantitative comparison of the cell surface expression of hDAT variants. Values represent the mean ± SEM of immunoreactive cell number, expressed as a percentage of total cell counts from 3 experiments. Statistical analyses were performed using Student's *t*-test. *P<0.05.

Fig. 6 shows the immunocytochemical analysis of the expression of FL hDAT and hDATΔEX6 in COS-7 cells treated with (permeabilized) or without (non-permeabilized) Triton using the anti-hDAT-EL2 antibody. Approximately 25% of Triton-treated cells expressing hDATΔEX6 exhibited immunoreactivity (Fig. 6A-a). Untreated cells expressing hDATΔEX6 showed similar results to the triton-treated cells (Fig. 6A-b), while untreated cells expressing FL hDAT showed no immunoreactivity (Fig. 6A-c).

**Figure 7:**
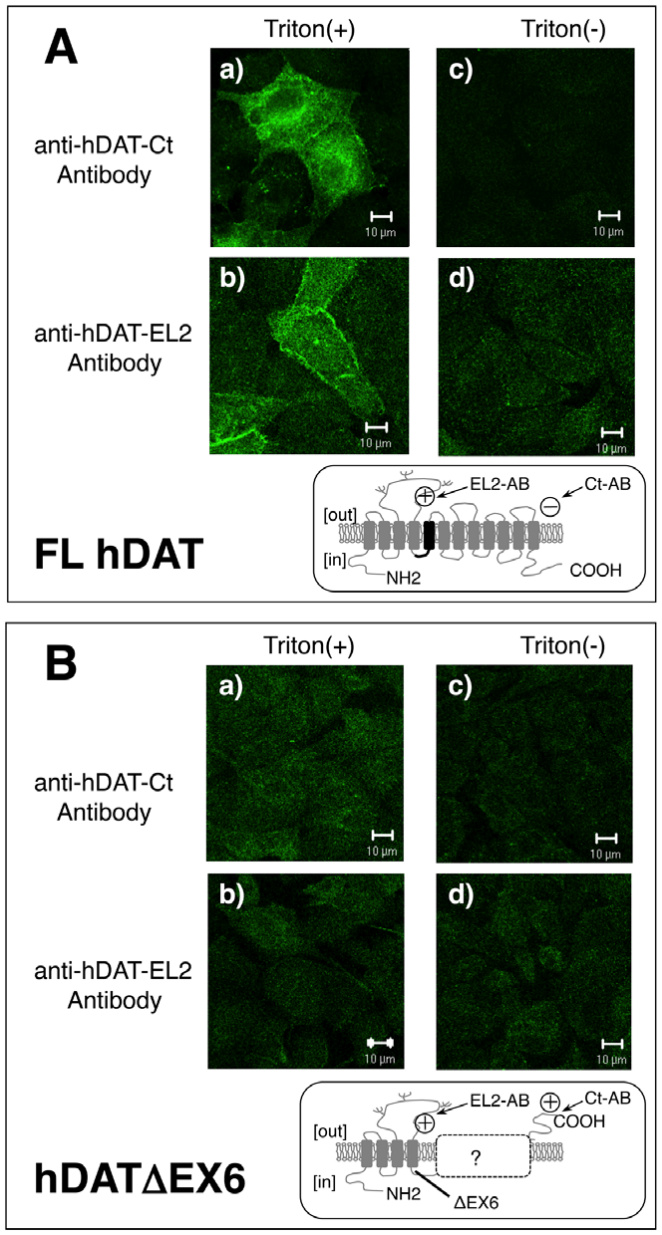
Cell surface expression of the DAT variant determined with two antibodies against different epitopes in stably transfected MDCK cells. MDCK cells stably expressing FL hDAT (**A**) and hDATΔEX6 (**B**) were treated with (a, b) or without (c, d) 0.25% Triton X-100 for 5 min, and then examined for the expression of DAT variants by confocal microscopy with immunological detection using anti-hDAT-Ct (a, c) and anti-hDAT-EL2 (b, d) antibodies. Each insert shows the predicted membrane topology of FL hDAT and hDATΔEX6, respectively.

To avoid the effects of transfection efficiency and cell-type specificity, we further analyzed the MDCK cells stably expressing FL hDAT and hDATΔEX6 using two antibodies, anti-hDAT-EL2 and anti-hDAT-Ct. The anti-hDAT-EL2 antibody (Chemicon, AB5802) recognizes an epitope of 18 amino acids (NH_2_-CHLHQSHGIDDLGPPRWQ) located in the 2nd extracellular loop, while the anti-hDAT-Ct antibody (Chemicon, AB1766) recognizes an epitope of 22 amino acids (NH_2_- CEKDRELVDRGEVRQFTLRHWL) in the intracellular C-terminal region. Among the MDCK cells expressing FL hDAT, immunoreactivity to the anti-hDAT-Ct antibody was only observed in those cells treated with Triton (Fig. 7A). Among the MDCK cells expressing hDATΔEX6, however, immunoreactivity to the anti-hDAT-Ct antibody was detected even in the untreated cells (Fig. 7B). Control experiments using the anti-hDAT-EL2 antibody showed immunoreactivity in the Triton-treated and untreated cells expressing both FL hDAT (Fig. 7A) and hDATΔEX6 (Fig. 7B). These results strongly suggest the C-terminus of hDATΔEX6 to be located extracellulary.

### Effects of co-expression with human DAT and NET variants

Recent observations suggested the dimerization of hDAT, which participates in cell surface expression [10]. To assess the possible interaction of FL hDAT and hDATΔEX6, or FL hNET and hNETΔEX6, we analyzed the functional expression of FL hDAT or FL hNET when co-transfected with the truncated isoforms.

**Figure 8:**
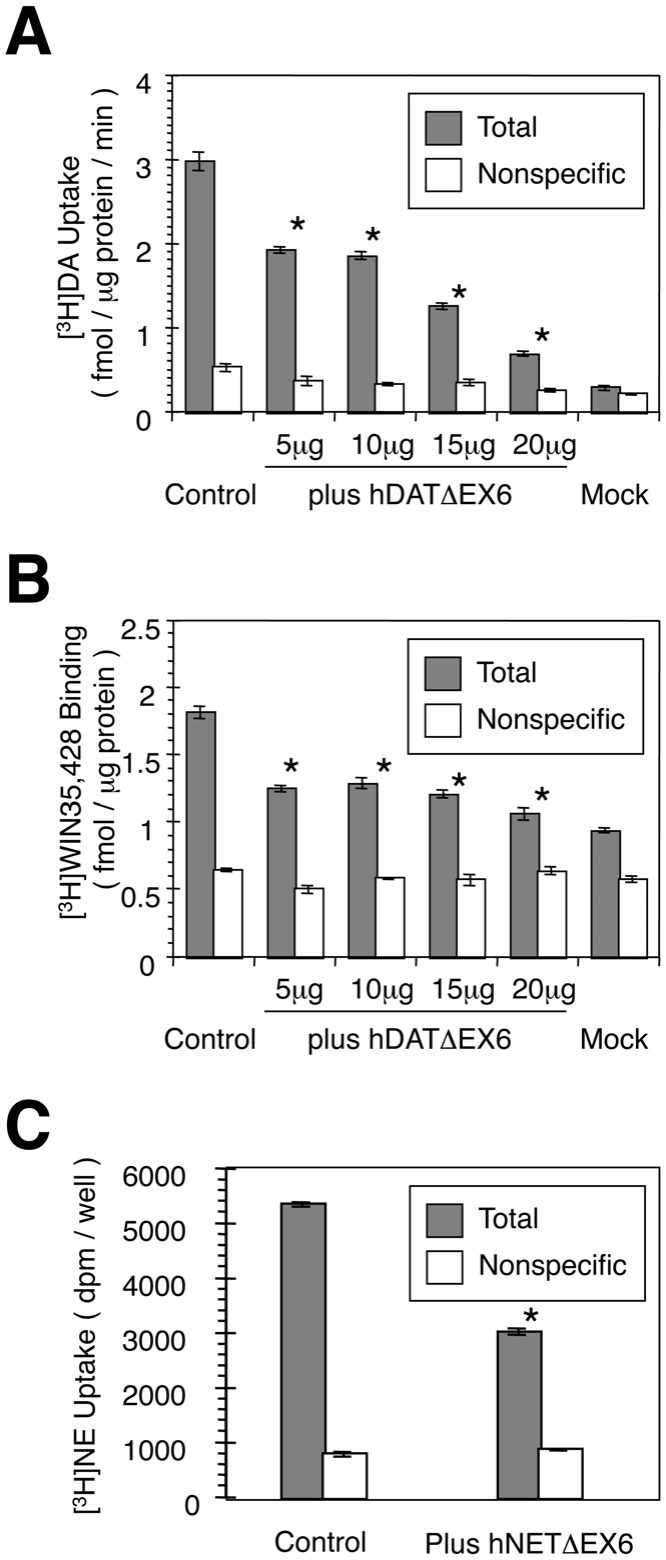
Dominant negative effect of the variant on the activity of FL hDAT and hNET in co-transfected COS-7 cells. **A, B.** COS-7 cells were transfected with 5 µg of FL hDAT cDNA alone (control) or together with various amounts of hDATΔEX6 cDNA. The total amount of DNA for transfection was adjusted using pcDNA3 to 25 µg. A [^3^H]DA (10 nM) uptake assay (A) and a [^3^H]WIN35,428 (2 nM) binding assay (B) were carried out in parallel in the presence (nonspecific) or absence (total) of 100 µM cocaine. Values represent the mean ± SEM for 3 experiments each performed in triplicate. *P<0.05 vs control. **C.** COS-7 cells were transfected with FL hNET alone (control) or together with hNETΔEX6, and subjected to a [^3^H]NE (10 nM) uptake assay. Nonspecific uptake was carried out in the presence of 10 µM nisoxetine. Values represent the mean ± SEM for 3 experiments each performed in triplicate. Statistical analyses were performed using an analysis of variance (ANOVA) with pair-wise comparisons by the Bonferroni method. *P<0.05 vs control.

Co-expression of FL-hDAT and hDATΔEX6 resulted in a significant reduction of [^3^H]DA uptake, as compared with that observed in the control cells transfected with FL hDAT cDNA and an empty vector (Fig. 8A). In parallel, co-expression of FL hDAT and hDATΔEX6 caused a significant reduction in the binding of a cocaine analogue, [^3^H]WIN35,428 (Fig. 8B). Similarly, co-expression of FL hNET and hNETΔEX6 resulted in a significant reduction of [^3^H]NE uptake, as compared with that observed in the control cells expressing FL hNET alone (Fig. 8C).

**Figure 9:**
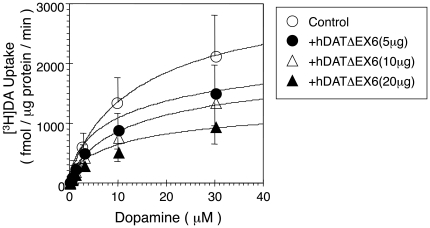
Kinetic analysis of the effect of hDATΔEX6 on FL hDAT activity. The effect of hDATΔEX6 on FL hDAT activity was analyzed kinetically in co-transfected COS-7 cells. COS-7 cells were transfected with FL hDAT alone (control) or together with various amounts of hDATΔEX6. The total amount of DNA was adjusted using pcDNA3 to 25 µg. Cells were incubated with 10 nM [^3^H]DA and various concentrations (0.1–30 µM) of unlabeled DA at 37°C for 6 min. Specific uptake was determined by subtracting nonspecific uptake in the presence of 100 µM cocaine. Values represent the mean ± SEM for 3 experiments each performed in triplicate.

A kinetic analysis of [^3^H]DA uptake revealed a significant decrease in V_max_ without an alteration in K_m_ (Fig. 9 and Table S1).

**Figure 10:**
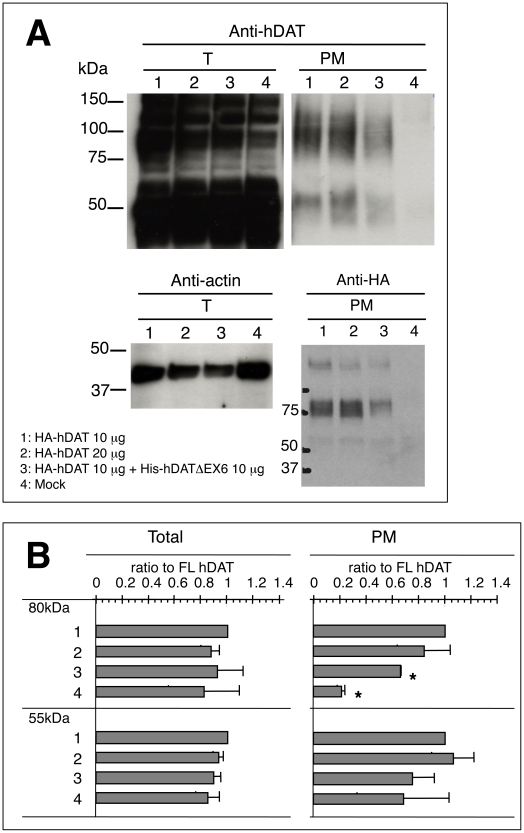
Co-expression of hDATΔEX6 diminished the cell surface expression of FL hDAT. **A.** COS-7 cells grown on 60 mm Petri dishes were transiently transfected with 2 µg (lane 1) or 4 µg (lane 2) of HA-hDAT alone, 2 µg of HA-hDAT with an equal amount of His-hDATΔEX6 (lane 3), or 2 µg of His-hDATΔEX6 alone (lane 4) using FuGENE6. The total amount of DNA was adjusted with pcDNA3 to 4 µg. Two days after transfection, cells were incubated with sulfo-NHS-SS-biotin for 60 min at 4°C, and then processed for the isolation of biotinylated proteins in the plasma membrane. Western blotting was performed with anti-hDAT-EL2 antibody for total (T) and biotynilated (PM) proteins (upper panel), and with anti-HA antibody for biotynilated (PM) proteins (lower right panel). The lower left panel shows the control using anti-actin antibody for total (T) protein. Data represent a typical result of repetitive experiments. **B.** Quantitative analysis of the effects of hDATΔEX6 on the cell surface expression of the mature (80 kDa) and immature (55 kDa) proteins. Absorbance was measured in the total fraction before NeutraAvidin precipitation (left, Total) and in NeutraAvidin precipitates (right, PM). Results are displayed as a ratio to the FL hDAT band's density, and values represent the mean ± SEM for three separate experiments. Statistical analyses were performed using Student's *t*-test. *P<0.01 vs control (FL hDAT).

A cell surface biotinylation assay with an immunoblot analysis using the anti-hDAT-EL2 antibody demonstrated a reduction in the expression of the 80–85 kDa hDAT at the cell surface, on co-expression with hDATΔEX6 (Fig. 10A). Since the anti-hDAT-EL2 antibody used here (and also the anti-hDAT-Ct antibody) recognizes both FL and truncated hDAT, we constructed an N-terminally HA-tagged FL hDAT (HA-hDAT) to explore the effect of hDATΔEX6 further in an immunoblot analysis using the anti-HA antibody. HA-hDAT exhibited a robust uptake of [^3^H]DA similar to FL hDAT (data not shown). The cell surface expression of HA-hDAT was reduced, on co-expression with hDATΔEX6 (Fig. 10A). Fig. 10B shows the results of quantitative analyses of the immunoblots using the anti-hDAT-EL2 antibody. Again, co-expression of hDATΔEX6 decreased the cell surface expression of the 80–85 kDa mature hDAT. These results were consistent with the decreased V_max_ of [^3^H]DA uptake.

**Figure 11:**
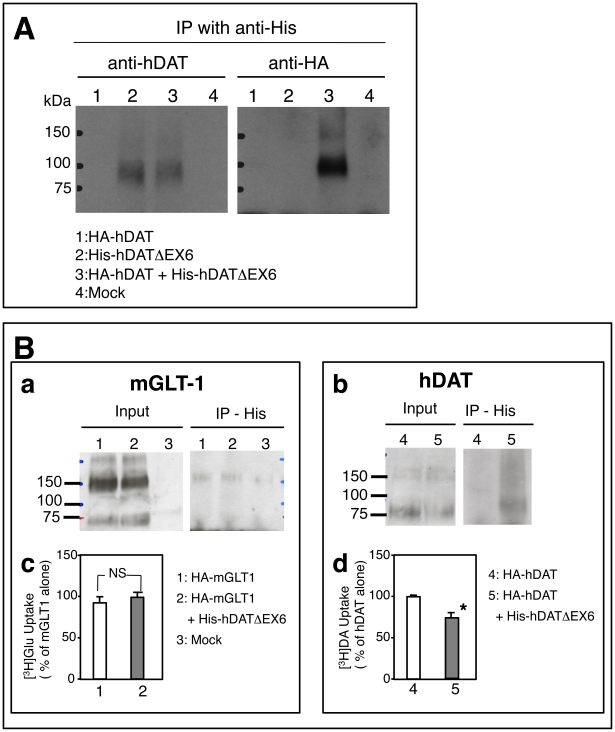
hDATΔEX6 physically interacts with FL hDAT. **A.** COS-7 cells grown on 60 mm Petri dishes were transfected with 2 µg of HA-hDAT alone (lane 1), 2 µg of His-hDATΔEX6 (lane 2), 2 µg of HA-hDAT with an equal amount of His-hDATΔEX6 (lane 3), or 4 µg of empty vector pcDNA3. The total amount of DNA was adjusted with pcDNA3 to 4 µg. Two days after transfection, HA-hDAT was co-immunoprecipitated with His-hDATΔEX6 using anti-His antibody. A western blot analysis of the immunoprecipitates obtained with the anti-His antibody was performed using anti-hDAT-EL2 antibody (left) and anti-HA antibody (right). The data represent a typical result from one experiment followed by 2 additional experiments with similar results. **B.** (**a, b**) COS-7 cells grown on 60 mm Petri dishes were transfected with 0.25 µg of HA-mGLT1 alone (lane 1), 0.25 µg of HA-mGLT1 with an equal amount of His-hDATΔEX6 (lane 2), 2 µg of empty vector pcDNA3 (lane 3), 0.25 µg of HA-hDAT alone (lane 4), or 0.25 µg of HA-hDAT with an equal amount of His-hDATΔEX6 (lane 5). The total amount of DNA was adjusted with pcDNA3 to 2 µg. Two days after transfection, HA-mGLT1 (a) or HA-hDAT (b) was co-immunoprecipitated with His-hDATΔEX6 using anti-His antibody. A western blot analysis of the immunoprecipitates obtained with the anti-His antibody was performed using anti-HA antibody. (**c, d**) In parallel experiments, COS-7 cells transfected with HA-mGLT1 alone (control) or together with His-hDATΔEX6 (c), or those transfected with HA-hDAT alone (control) or together with His-hDATΔEX6 (d) were subjected to uptake assays for [^3^H]glutamic acid (10 nM) and [^3^H]DA (10 nM), respectively. Values represent the mean ± SEM for three experiments each performed in duplicate, and are expressed as a percentage of HA-mGLT alone (9497±960 dpm/well/10 min) or HA-hDAT alone (42769±5903 dpm/well/10 min). Statistical analyses were performed using the Mann-Whitney *U*-test. *P<0.05 vs control.

Interaction among the isoforms was further examined by conducting immunopreciptation assays using tagged forms of hDAT, such as HA-hDAT and N-terminally His-tagged hDATΔEX6 (His-hDATΔEX6). The proteins precipitated with the anti-His antibody were subjected to an immunoblot analysis using the anti-hDAT-EL2 antibody and anti-HA antibody (Fig. 11A). Isolation of His-hDATΔEX6 with the anti-His antibody allowed the detection of HA-hDAT using the anti-HA antibody when the two proteins were expressed simultaneously, indicating that FL hDAT and hDATΔEX6 form heterooligomeric complexes.

To exclude the possibility of nonspecific aggregation with other membrane proteins including neurotransmitter transporters, we performed additional immunoprecipitation assays using an HA-tagged mouse glutamate transporter GLT-1 (HA-mGLT-1) and His-hDATΔEX6 (Fig. 11B), according to our previous study on the interaction of rat NET splice variants with the wild-type transporters including NET, GAT, and GLAST [11]. Consistent with previous observations [22], HA-mGLT-1 migrated as a monomeric band (∼75 kDa) and also as multimers (∼150 kDa) on SDS-PAGE (Fig. 11B_a, Input). Immunoprecipitation with anti-His antibody did not detect HA-mGLT-1 in COS-7 cells co-transfected with His-hDATΔEX6 (Fig. 11B_a, IP-His), in association with the lack of an effect on [^3^H]glutamate uptake through mGLT-1 (Fig. 11B_c). In parallel experiments, the interaction of FL hDAT with hDATΔEX6 was confirmed by immunoprecipitation (Fig. 11B_b) and uptake assays (Fig. 11B_d). Taken together, it is conceivable that hDATΔEX6 interacts with FL-hDAT specifically.

## Discussion

There is an increasing body of evidence suggesting an important role for alternative splicing in regulation of the expression and function of Na^+^- and Cl^−^-dependent neurotransmitter transporters including the glycine transporter [23], [24] and NET [6], [21], [25], [26]. Previous studies have demonstrated several splice variants of NET, differing in expression and function [6]. These isoforms differ in the C-terminal region, and revealed unique features of expression and function [21], [25], [26]. In the present study, we found novel variants of hDAT and hNET, which lacked the region encoded by exon 6, resulting in a truncated protein. Therefore, we further examined their expression and function.

hDATΔEX6 was located mostly in the cytosolic compartment with some expression at the cell surface in the transiently transfected COS-7 cells and in the stably transfected MDCK cells. The cell surface expression of hDATΔEX6 increased gradually after transfection, reaching a maximum at 3 days, while the total protein level was constant after the transfection of COS-7 cells. The slow delivery of hDATΔEX6 protein to the plasma membrane might underscore its cytosolic retention. A slow delivery to the plasma membrane was also observed in the C-terminal mutant DATs [27].

The hDATΔEX6 protein was observed in the plasma membrane. However, no transport activity was found in the cells transfected with hDATΔEX6 cDNA even at later periods, suggesting that hDATΔEX6 is an inactive isoform in the plasma membrane. Recently, Yamashita et al. [9] analyzed the structure of LeuT, a bacterial homologue of the mammalian Na^+^- and Cl^−^-dependent neurotransmitter transporter, using X-ray crystallography. They demonstrated that the 1st and 6th TMDs associated to produce a binding pocket for substrates and Na^+^ in the middle of each TMD. Removal of the 5th TMD in hDATΔEX6 likely affected the interaction between the 1st and 6th TMDs, leading to a loss of the transport function.

Another important finding of the present study was the unique membrane topology of hDATΔEX6. An immunocytochemical analysis using two antibodies specific for DAT but recognizing intra- and extracellular regions suggested the C-terminus of hDATΔEX6 to be located extracellulary. Deletion of the region encoded by exon 6 in the hDATΔEX6 variant is believed to affect the orientation of those TMD regions that follow. The present findings, suggesting the C-terminal region to be located extracellularly, did not indicate the exact membrane topology of each TMD including the 7–12th TMDs. A previous study suggested an alternative membrane topology consisting of TMD in the EL2 region corresponding to the involvement of N-terminal regions TM1 and TM2 [28]. The predicted membrane topology of hDATΔEX6 indicated in Fig. 7 was simply reflected by the sequence of hydrophobic regions considered as TMDs. It is of great interest to determine the precise membrane topology of the hDATΔEX6 variant.

The mechanism underlying the changes in membrane trafficking of hDATΔEX6 remains unclear at present. Studies with DAT mutants have suggested several possibilities, including (1) an elimination of phosphorylation in the C-terminal region by PKC as observed for other neurotransmitter transporters [29], (2) a loss of interaction with proteins such as PICK1 in the PDZ domain at the C-terminal end [30], and (3) an unknown mechanism independent of interaction with the PDZ protein at the C-terminal [31]. Studies on C-terminus splice variants of hNET also documented a critical contribution of the hNET C-terminus to transporter trafficking, stability, and function [21], [25], [26]. These explanations seem unlikely in the case of hDATΔEX6, since the present findings suggested the C-terminus of hDATΔEX6 to be located extracellulary. At present, it is unknown how trafficking of hDATΔEX6 to the plasma membrane is regulated, and further study is needed to clarify this.

We also found that hDATΔEX6 had a dominant negative effect on FL hDAT, possibly through the formation of heterooligomeric complexes, as suggested by the results of the immunoprecipitation assays. There is a growing body of evidence that oligomerization is necessary for the cell surface expression of neurotransmitter transporters including DAT [10], [27]. The structure of LeuT suggested dimerization through interaction at the 9th and 12th TMDs [9]. However, it seems unlikely that FL hDAT and hDATΔEX6 interact in these regions, since according to the predicted membrane topology of hDATΔEX6, the C-terminal region is located extracellulary, preventing direct interaction. Torres et al. [10] found that the C-terminal of DAT was not essential for oligomerization, and that a small fragment comprising the first two TMDs inhibited the wild-type transporter function but not when the leucine repeat motif present in the 2nd TMD was mutated. However, it is unclear wether FL hDAT and hDATΔEX6 associate in the same way, since immunoprecipitation assays do not reveal modes of interaction and no information is available about the membrane topology of hDATΔEX6 except the C-terminus. Therefore, further study is needed to determine the mechanism by which hDATΔEX6 forms a heterooligomeric complex with FL hDAT.

An alternative explanation for the mechanism underlying the dominant negative effect of the splice variant is that hDATΔEX6 interacts with FL hDAT at the plasma membrane to produce a dominant negative effect on the activity of FL hDAT. The present finding that part of the hDATΔEX6 protein was observed in the plasma membrane may support this possibility. However, there is evidence that the functional unit of the transporter is a monomer, though isoforms or different transporters such as NET and SERT consist of heterodimers [32]. Further study is needed to clarify this possibility.

The splicing of DAT and NET transcripts might have physiological and pathophysiological relevance to catecholaminergic neurons in both the central and peripheral nervous system, since the present findings indicate that splicing affects the expression and function of catecholamine transporters. Although the expression of hDATΔEX6 was observed at the mRNA level, that at the protein level was observed only in the expression systems, and we do not have proof yet of protein expression in vivo. Disease-associated changes of DAT and NET expression have been observed in peripheral blood cells [33], [34], suggesting the cells to be a potential marker for some CNS disorders including Parkinson's disease. The expression of splice variants in these cells may contribute to the development of new diagnostic markers for such disorders, although a preliminary examination failed to reveal the expression of hDATΔEX6 in the brains of PD patients.

In summary, the present study demonstrated that the novel splice variant hDATΔEX6 and probably hNETΔEX6 had a unique membrane topology and a dominant negative effect on FL hDAT or FL hNET possibly through the formation of heterooligomeric complexes. The unique features of the variants observed here suggest the importance of isoforms of catecholamine transporters in monoaminergic signaling in the brain and peripheral tissues.

## Materials and Methods

### Materials

Drugs used in this study were dopamine hydrochloride, norepinephrine tartrate, pargyline hydrochloride (Nacalai Tesque, Inc., Kyoto, Japan) and nisoxetine hydrochloride (Sigma-Aldrich Corporation, St. Louis, MO, USA). [^3^H]DA (1176.6 GBq/mmol), [^3^H]NE (886–1480 GBq/mmol), [^3^H]Glutamic acid (1.835 TBq/mmol) and [^3^H]WIN35,428 (2.22 TBq/mmol) were purchased from PerkinElmer Life Sciences, Inc. (Boston, MA). RivatraAce and KOD-plus DNA polymerase were obtained from TOYOBO (Tokyo, Japan), restriction enzymes from New England Biolabs (Ipswich, MA), FuGENE6 transfection reagent from Roche Diagnostics (Mannheim, Germany), and DNA purification kits from Qiagen (Tokyo, Japan). Human placental total RNA was purchased from Ambion (Austin, TX) and Cell Applications, Inc. (San Diego, CA), human adult whole brain total RNA from Life Technologies (Gaithersburg, MD), fetal whole brain total RNA from Stratagene (La Jolla, CA), human substantia nigra total RNA of Parkinson's disease patients and normal subjects from Clontech (Palo Alto, CA), and cDNA from human adult adrenal, cerebral cortex, pons, cerebellum, medulla oblongata and placenta from Biochain Institute, Inc. (Hayward, CA). The rabbit anti-hDAT-EL2 antibody AB5802 and rabbit anti-hDAT-Ct antibody AB1766 were purchased from Chemicon (Temecula, CA), anti-human NET mouse monoclonal antibody NET17-1 from MAb Technologies (Stone Mountain, GA), anti-HA antibody from MBL (Nagoya, Japan), and anti-His antibody from Clontech. Sulfo-NHS-SS-biotin [sulfosuccinimidyl-2-(biotinamido)ethyl-1,3-dithiopropionate] and NeutrAvidin® beads were obtained from Pierce (Rockford, IL).

### Cloning of human DAT cDNA

The initial cloning of full-length cDNA of human DAT was performed by RT-PCR in three discrete and overlapping regions with a pool of first strand cDNA synthesized from the total RNA of white blood cells. PCR was performed with initial denaturation at 92°C for 2 min, followed by 40 cycles of 92°C for 30 sec and 68°C for 4 min using specific primers, hDAT-P#3 (5′-ATGGTACCAGAATTCCTCAACTCCCAGTGTGCCCATG; 5′-region upstream of ATG translation initiation site)/hDAT-P#2 (5′-GTACTCGGCAGCAGGTGTGGTC) for the 5′-region, hDAT-P#4 (5′-GCTTCACGGTCATCCTCATCTCACTG)/hDAT-P#6 (5′-CTGCTGGATGTCGTCGCTGAACTG) for the downstream region and hDAT-P#5 (5′-GCTGCACAGACACCGTGAGCTCTTCAC)/hDAT-P#7 (5′-ATCTCGAGTCTTCGTCTCTGCTCCCTCTACAC; 3′-region downstream of translation stop codon at exon 15) for the 3′-region. The products were isolated from agarose gel, digested with KpnI, BanHI, NgoMIV or XhoI, and subcloned into pcDNA3 at the KpnI/XhoI digestion site. Each clone was subjected to restriction enzyme digestion and nucleotide sequencing.

### Analysis of mRNA expression

The expression of hDAT and hNET mRNA variants produced by alternative splicing was analyzed by RT-PCR using primers specific for each variant. Total RNA was isolated from white blood cells prepared from whole blood of healthy human volunteers by the acid-phenol method [35] using Isogen. Other human total RNA was obtained from commercial sources. First strand cDNA from 1–10 µg of total RNA was synthesized with random hexamer primers and RNaseH(-)-MMLV reverse transcriptase (RivatraAce). PCR was performed with initial denaturation at 92°C for 2 min, followed by 35 cycles of 92°C for 30 sec, 62°C for 30 sec and 72°C for 2 min (for hDAT) or 35 cycles of 92°C for 30 sec, 55°C for 30 sec and 68°C for 2 min (for hNET) with a final extention at 72 or 68°C for 5 min using Kod-Plus. The primers used were: 5′-CGAGTACTTTGAACGTGGCGTGCTGCAC (hDAT-P#11)/5′-CTTGTTGTAGCTGGAGAAGGCGATCAGC (hDAT-P#12) for hDAT and 5′-CTACTACAACGTCATCATCGCC (hNET-P#17)/5′-AGATGGCGAACCCAGAGACG (hNET-P#21) for hNET. The PCR products were analyzed by electrophoresis on agarose gel. The amplicons were isolated from the gel, and then sequenced directly or after subcloning into the pGEM-T Easy plasmid vector using PCR primers or T7 and SP6 primers, respectively, to confirm the splicing.

### Cell preparation and transfection

COS-7 cells and MDCK cells (RIKEN Cell Bank, Tsukuba, Japan) were maintained in Dulbecco's modified Eagle's medium (DMEM) supplemented with 10% fetal calf serum, 100 units/ml penicillin-G, 100 µg/ml streptomycin and 2.5 µg/ml fungisone at 37°C under 5% CO_2_/95% air.

For uptake assays, COS-7 cells at subconfluence were harvested and transfected with pcDNA3 alone or with pcDNA3 containing hDAT, hNET, or mGLT1 cDNA and/or variant cDNA by electroporation or using FuGENE6 according to the manufacturer's directions [21], [36]. mGLT-1 cDNA (AK134609) in the plasmid vector pFLC1 was obtained from RIKEN Mouse FANTOM FLS through KK DNAFORM [37], and a BamHI fragment containing ORF was subcloned into pcDNA3. Cells were diluted in culture medium, plated in 24-well tissue culture plates and cultured for 2–3 days.

For immunological analyses, COS-7 cells at subconfluence in 60-mm diameter Petri dishes were transfected with 4 µg of DNA using FuGENE6. For stable transfections, MDCK cells were transfected using FuGENE6 and selected using G418 [21].

### Uptake and binding assays

Cells were washed three times with an oxygenated Krebs Ringer HEPES-buffered solution (KRH; 125 mM NaCl, 5.2 mM KCl, 1.2 mM CaCl_2_, 1.4 mM MgSO_4_, 1.2 mM KH_2_PO_4_, 5 mM glucose, and 20 mM HEPES, pH 7.3±0.1) and incubated for 10 min at 37°C with 10 nM [^3^H]DA, 10 nM [^3^H]NE, or 10 nM [^3^H]glutamic acid, as described previously [21], [36]. For assays of DA and NE uptake, 0.1 mM ascorbate and 50 µM pargyline were added to the solution. After the removal of excess radioligands, cells were washed rapidly three times with ice-cold KRH. Radioactivity remaining in the cells was extracted with NaOH and measured by liquid scintillation spectrometry. Nonspecific uptake was determined from values obtained for cells transfected with pcDNA3 alone and from results of uptake assays performed in the presence of 10 µM GBR12935 or nisoxetine, 100 µM cocaine, or 10 mM unlabeled glutamic acid. For kinetic analyses, cells were incubated in KRH containing 10 nM [^3^H]DA and 0.1–30 µM unlabelled DA.

For binding assays, cells were incubated with 2 nM [^3^H]WIN35,428 in KRH buffer at 4°C (on ice) for 2 h in the absence (total binding) or presence (nonspecific binding) of 1 mM DA [36].

Kinetic analyses of uptake data and Eadie-Hofstee plots were conducted using Prism 3 (GraphPad Softwares, San Diego, CA). Statistical analyses were performed using an analysis of variance (ANOVA) with pair-wise comparisons by the Bonferroni method.

### Immunocytochemistry and confocal microscopy

For immunostaining experiments, MDCK cells were grown on Costar Transwell® filter supports (Corning, Acton, MA) at 2×10^5^ cells/well. The cells were initially rinsed with Ca^2+^- and Mg^2+^-containing PBS, and then fixed in 4% paraformaldehyde. After three washes with PBS, cells were permeabilized in PBS containing 0.25% Triton X-100 for 5 min, and incubated in blocking solution (2% goat serum) for 30 min. Cells were incubated with a rabbit anti-hDAT polyclonal antibody (anti-hDAT-Ct antibody (1∶500) or anti-hDAT-EL2 antibody (1∶250)) overnight at 4°C, followed by a FITC-conjugated anti-rabbit secondary antibody. Cells were then washed three times with PBS, and the filter with cells was excised from its support and mounted on a glass slide with Perma Fluor® aqueous mounting medium (Thermo Shandon, Pittsburgh, PA). Immunostaining of COS-7 cells grown on Falcon Biocoat® culture slides (Becton Dickinson Labware, Bedford, MA) was performed as above. After a final wash, the cells were covered with a coverslip and mounting medium. Immunofluorescent images were generated using a Zeiss laser scanning confocal microscope (LSM510, Central Research Laboratory, Okayama University Medical School). The z-series (for MDCK cells) was collected by focusing initially on the middle section of the cells (position 0) and scanning 6 µm above to 6 µm below this plane at 0.2 µm intervals.

### Cell surface Biotinylation and Western blotting

Cells were washed with ice cold Ca^2+^- and Mg^2+^-containing PBS and incubated for 1 h at 4°C with 1 mg/ml sulfo-NHS-SS-biotin. Cells were then washed with PBS, collected and solubilized in RIPA buffer, and incubated at 4°C for 30 min. Non lysates were removed by centrifugation for 20 min at 12000 rpm, and an aliquot of each sample was used for the isolation of biotinylated proteins with NeutrAvidin® beads by incubating overnight at 4°C with gentle shaking. The precipitated proteins were then washed five times with RIPA buffer, and denatured by heating the beads in sample buffer at 95°C for 5 min. Samples were analyzed by SDS-PAGE (5–20% gel, BioRad, Tokyo, Japan), and transferred onto a PVDF membrane (GE Healthcare Biosciences, Buckinghamshire, UK). Western blotting was performed with polyclonal rabbit antibodies to hDAT or a monoclonal mouse antibody to hNET followed by an HRP-conjugated secondary antibodies, and detected on autoradiographic film. The quantification of signals was performed using densitometry and NIH Image software, as described [21]. Statistical analyses were performed using Student's *t*-test.

### Immunoprecipitation

Cells co-expressing HA-hDAT and His-hDATΔEX6, or those co-expressing HA-mGLT1 and His-hDATΔEX6 were homogenized with RIPA buffer, and the lysates were subjected to immunoprecipitation overnight at 4°C with 0.5 µg of anti-His antibody, followed by 1 h of incubation with a 50% slurry of protein-A Sepharose beads (GE Healthcare Biosciences). Beads were washed four times with RIPA buffer, and the proteins bound to the beads were eluted with loading buffer and subjected to SDS-PAGE, as described above. Immunoblot analyses of the precipitates were carried out using the anti-hDAT-EL2 antibody and anti-HA antibody.

## Supporting Information

Figure S1 Schematic representation of the organization of the human DAT and NET genes (A), and comparison of DAT/NET genes at the exon 6 - intron5 boundary (B). A. Boxes and bars represent exons and introns, respectively. A translation initiation codon exists in exon 2. The arrow in exon 6 represents an alternative splicing site. Insert shows a scheme of the alternative splicing. The arrowhead indicates positions of the primers used. B. The position of exon 6 (capital letters) was assigned based on the consensus AG-GT (underlined) in the adjacent introns 5 and 6 (small letter). The pyrimidine-rich consensus sequence for splicing is shaded, a possible exonic splicing enhancer (ESE) motif for the SR protein SF2/ASF is underlined by a dotted line, and the GA-rich ESE motif is boxed.
(0.86 MB TIF)

Figure S2 RT-PCR analysis of the expression of the hDAT variant in Substantia nigra from normal subjects and Parkinson disease patients. Total RNA from human Substantia nigra of patients with Parkinson's disease (PD) or normal subjects (Cont) was obtained commercially, and used to synthesize first strand cDNA (RT+, with RivatraAce; RT-, without RivatraAce). PCR was performed with initial denaturation at 94°C for 2 min, followed by 40 cycles of 92°C for 30 sec and 68°C for 2 min with a final extention at 68°C for 5 min using Kod-Plus. The primers used were: 5′-CGAGTACTTTGAACGTGGCGTGCTGCAC (hDAT-P#11)/5′-GTGGTGACAATCGCGTCCCTGTAGCAG (hDAT-P#10). The PCR products were analyzed by electrophoresis on agarose gel. C: FL hDAT, V: hDATΔEX6, N: negative control (water as a template), M: DNA marker of 100 bp.
(0.66 MB TIF)

Table S1 Kinetic analysis of the effect of hDATΔEX6 on hDAT activity in co-transfected COS-7 cells. COS-7 cells were transfected with the full-length (FL) hDAT alone (control) or with various amounts of the splice variant hDATΔEX6. The total amount of DNA for transfection was adjusted with pcDNA3 to 25 µg. Uptake assays were carried out by incubating cells with 10 nM [3H]dopamine in the presence of various concentrations (0.1–30 µM) of unlabelled DA at 37°C for 6 min. Specific uptake was determined by subtracting the nonspecific uptake measured in the presence of 100 µM cocaine. Values represent the mean ± SEM for 3 experiments each performed in triplicate. Vmax was expressed as a ratio to the control (FL hDAT alone) value, which was 2.03±0.55 fmol/µg protein/min. *Significantly different from control at P<0.05.
(0.03 MB DOC)
