# Supplementary figures and images for: Expression and Function of Variants of Human Catecholamine Transporters Lacking the Fifth Transmembrane Region Encoded by Exon 6
Source: PLoS One. 2010 Aug 5;5(8):e11945. doi: 10.1371/journal.pone.0011945 (PMC2916826; doi:10.1371/journal.pone.0011945)

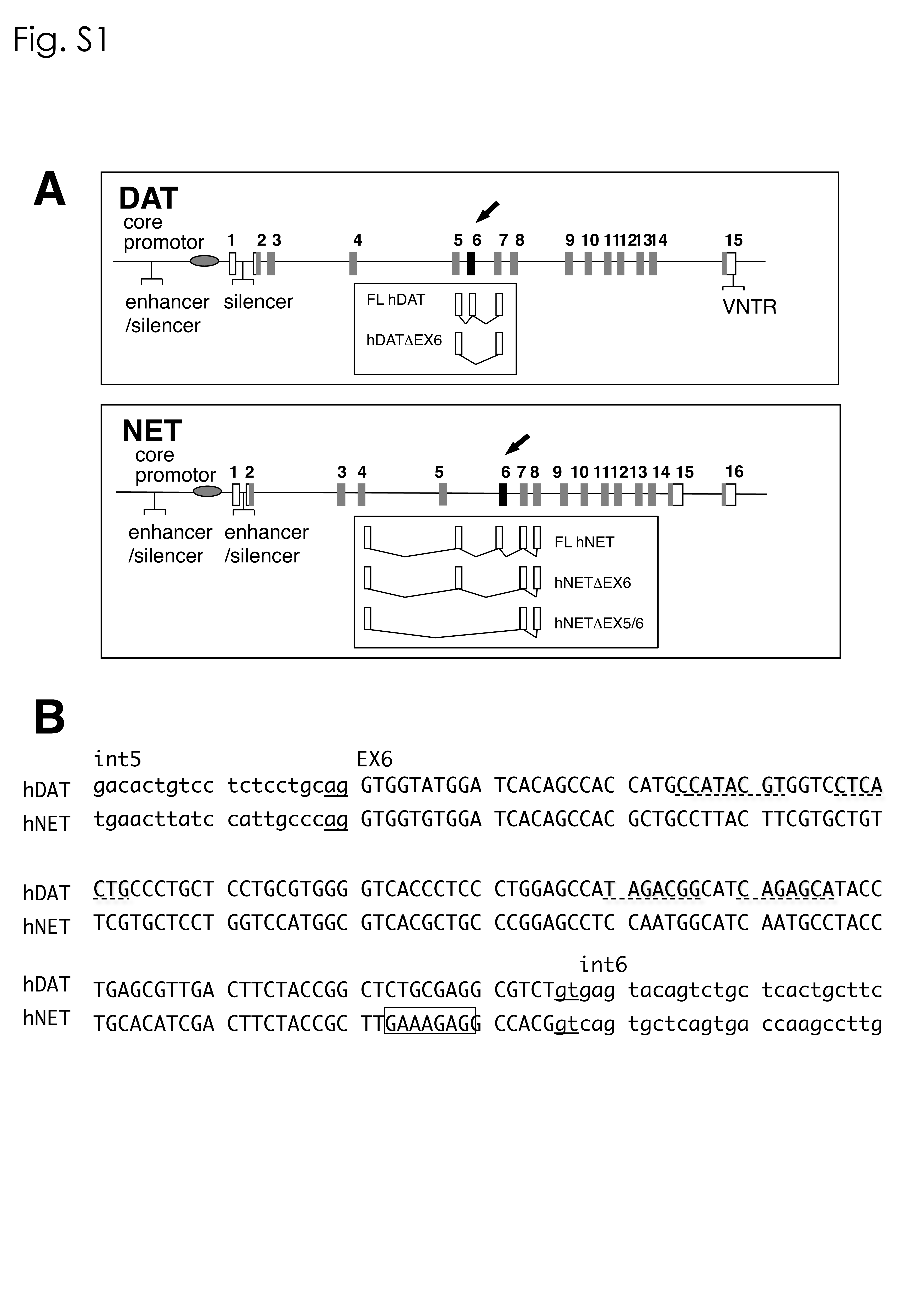

Supplement: Figure S1 — Schematic representation of the organization of the human DAT and NET genes (A), and comparison of DAT/NET genes at the exon 6 - intron5 boundary (B). A. Boxes and bars represent exons and introns, respectively. A translation initiation codon exists in exon 2. The arrow in exon 6 represents an alternative splicing site. Insert shows a scheme of the alternative splicing. The arrowhead indicates positions of the primers used. B. The position of exon 6 (capital letters) was assigned based on the consensus AG-GT (underlined) in the adjacent introns 5 and 6 (small letter). The pyrimidine-rich consensus sequence for splicing is shaded, a possible exonic splicing enhancer (ESE) motif for the SR protein SF2/ASF is underlined by a dotted line, and the GA-rich ESE motif is boxed. (0.86 MB TIF) [file pone.0011945.s001.tif]

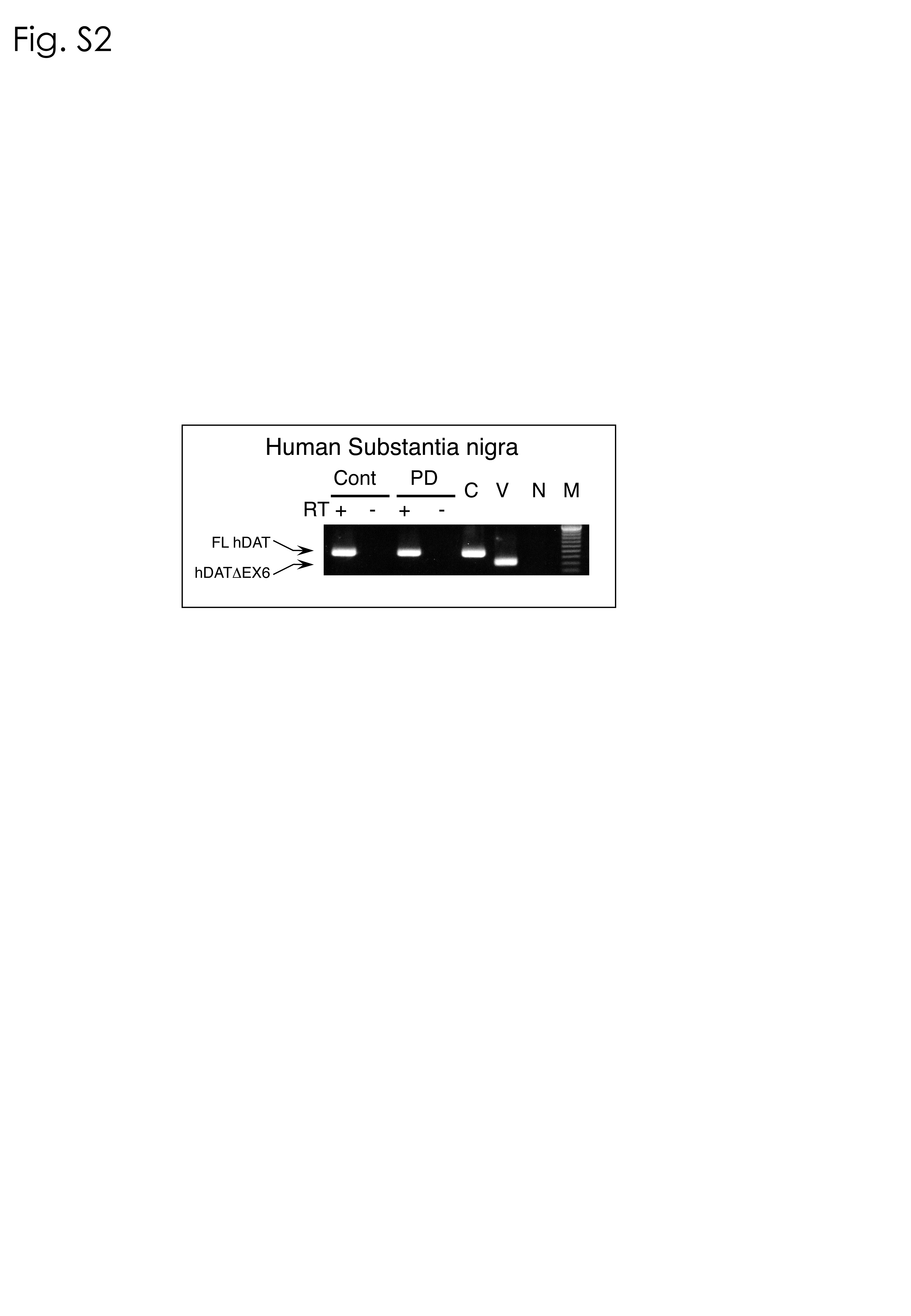

Supplement: Figure S2 — RT-PCR analysis of the expression of the hDAT variant in Substantia nigra from normal subjects and Parkinson disease patients. Total RNA from human Substantia nigra of patients with Parkinson's disease (PD) or normal subjects (Cont) was obtained commercially, and used to synthesize first strand cDNA (RT+, with RivatraAce; RT-, without RivatraAce). PCR was performed with initial denaturation at 94°C for 2 min, followed by 40 cycles of 92°C for 30 sec and 68°C for 2 min with a final extention at 68°C for 5 min using Kod-Plus. The primers used were: 5′-CGAGTACTTTGAACGTGGCGTGCTGCAC (hDAT-P#11)/5′-GTGGTGACAATCGCGTCCCTGTAGCAG (hDAT-P#10). The PCR products were analyzed by electrophoresis on agarose gel. C: FL hDAT, V: hDATΔEX6, N: negative control (water as a template), M: DNA marker of 100 bp. (0.66 MB TIF) [file pone.0011945.s002.tif]
