# Supplementary material for: Expression and Function of Variants of Human Catecholamine Transporters Lacking the Fifth Transmembrane Region Encoded by Exon 6
Source: PLoS One. 2010 Aug 5;5(8):e11945. doi: 10.1371/journal.pone.0011945 (PMC2916826; doi:10.1371/journal.pone.0011945)
Supplement: Table S1 — Kinetic analysis of the effect of hDATΔEX6 on hDAT activity in co-transfected COS-7 cells. COS-7 cells were transfected with the full-length (FL) hDAT alone (control) or with various amounts of the splice variant hDATΔEX6. The total amount of DNA for transfection was adjusted with pcDNA3 to 25 µg. Uptake assays were carried out by incubating cells with 10 nM [3H]dopamine in the presence of various concentrations (0.1–30 µM) of unlabelled DA at 37°C for 6 min. Specific uptake was determined by subtracting the nonspecific uptake measured in the presence of 100 µM cocaine. Values represent the mean ± SEM for 3 experiments each performed in triplicate. Vmax was expressed as a ratio to the control (FL hDAT alone) value, which was 2.03±0.55 fmol/µg protein/min. *Significantly different from control at P<0.05. (0.03 MB DOC) [file pone.0011945.s003.doc]

**Table S1. Kinetic analysis of the effect of hDATΔEX6 on hDAT activity in co-transfected COS-7** cells.

|  | Km (μM) | Vmax (ratio to control#) |
| --- | --- | --- |
| Control | 6.73 ± 0.90 | 1.00 |
| + 5μg hDATΔEX6 | 5.81 ± 1.00 | 0.710 ± 0.059* |
| + 10μg hDATΔEX6 | 6.08 ± 0.63 | 0.643 ± 0.041* |
| + 20μg hDATΔEX6 | 5.33 ± 0.56 | 0.389 ± 0.018* |

COS-7 cells were transfected with the full-length (FL) hDAT alone (control) or with various amounts of the splice variant hDATΔEX6. The total amount of DNA for transfection was adjusted with pcDNA3 to 25 μg. Uptake assays were carried out by incubating cells with 10 nM [^3^H]dopamine in the presence of various concentrations (0.1-30 μM) of unlabelled DA at 37 °C for 6 min. Specific uptake was determined by subtracting the nonspecific uptake measured in the presence of 100 μM cocaine. Values represent the mean ± SEM for 3 experiments each performed in triplicate. Vmax was expressed as a ratio to the control (FL hDAT alone) value, which was 2.03 ± 0.55 fmol/μg protein/min. *Significantly different from control at P<0.05.
